# Programmed Cell Death Ligand 1 Expression in Cytological and Surgical Non–Small Cell Lung Cancer Specimens in Association with EGFR Mutation and Overall Survival: A Single-Institution Experience

**DOI:** 10.5146/tjpath.2022.01572

**Published:** 2022-09-15

**Authors:** Elif Sayman Gokal, Fugen Vardar Aker, Zuhal Kus Sılav, Bala Basak Oven

**Affiliations:** Department of Pathology, Inonu University, Faculty of Medicine, Malatya, Turkey; University of Health Sciences, Haydarpasa Numune Training and Research Hospital, Istanbul, Turkey; Department of Medical Oncology, BAU Medical Park Göztepe Hospital, Istanbul, Turkey

**Keywords:** Epidermal growth factor receptor, Non-small cell lung cancer, Programmed cell death ligand-1, Survival

## Abstract

***Objective:*** The aim of this study was to evaluate programmed cell death ligand-1 (PD-L1) expression and the relationship between driver mutations and survival analysis in advanced-stage non-small cell lung carcinoma (NSCLC).

***Material and Method:*** A total of 122 advanced-stage NSCLC patients were included in this retrospective study. The patients were diagnosed based on cytological examination and histopathological analysis of biopsy or resection material that had undergone at least 1 molecular analysis. The expression of PD-L1 in tumors and tumor-infiltrating lymphocytes (TIL) was scored and compared with age, sex, organ, biopsy method, tumor subtype, driver mutation status, and overall survival data.

***Results:*** There was no statistically significant difference between PD-L1-positivity and age, gender, location, pattern, or pathological diagnosis of the type of sample. When the threshold value for PD-L1 IHC evaluation was accepted as ≥1% and ≥50%, the rate of positivity was 19.7% and 7.4%, respectively.

***Conclusion:*** Since there is a wide range of positivity rates reported in the literature, we could not reach a conclusion as to whether the PD-L1-positivity rate we observed was high or low. There is a need for comparative studies where the technique, clones, threshold values, and phases are homogenized. There is an inverse correlation between the EGFR-mutant population and PD-L1 positivity. In terms of overall survival, no relationship was found between PD-L1 positivity, the presence of TIL, and EGFR mutation status.

## INTRODUCTION

Lung cancer is the leading cause of cancer-related deaths. Despite advances in surgical treatment, systemic therapy, and radiotherapy, the survival rate for all patients does not exceed 15% to 20%. More than half of patients are thought to be metastatic at the time of diagnosis, and the survival rate of advanced patients is reported to be much lower (1).**

**At the heart of newly developed treatment strategies in lung cancer, especially for non-small cell carcinoma (NSCLC), are some biomarkers that detect certain molecular changes, and which may provide individualized treatment options. Immune checkpoint blockade and anti-tumor immunity activation other than targeted therapies are also promising approaches that have become more important in recent years. Research in this field has gained momentum, and as a result immune checkpoint inhibitors were discovered to be one of the main mechanisms through which the tumor avoids the immune response. Two important immune checkpoints with established effectiveness in cancer treatment are the cytotoxic T-lymphocyte-associated antigen-4 pathway and the programmed cell death ligand-1 (PD-L1) (2).

## MATERIAL and METHOD

This study was approved by the ethics committee of the University of Health Sciences Faculty of Medicine, Haydarpasa Numune Training and Research Hospital (date: December 11, 2017, decision #: HNEAH-KAEK 2017/136).

### Selection of Cases and Data Collection

Cases diagnosed with advanced NSCLC based on cytological cell block, biopsy, or resection material analysis and that had undergone at least 1 molecular examination (*EGFR, ALK*, or *ROS1 *mutation analysis) during the study period (8 January 2013-7 January 2017) were scanned from the electronic, slide and block archives of our hospital. The age and gender of the patients, the date of diagnosis, biopsy site, the sampling method of the material (resection, small biopsy, fine-needle aspiration biopsy [FNAB], effusion, cell blocks), mutation status (*EGFR, ALK, ROS1*), and survival time were documented from the hospital database records. Once the case numbers were determined, the slides and paraffin blocks were removed from the archives and re-evaluated. Each case was diagnosed using at least 2 immune biomarkers (thyroid transcription factor-1 and p63/p40). Blocks suitable for immunohistochemical analyses were selected. The cell block slides and small biopsies containing more than 100 tumor cells were included.

### Preparation of the Slides and Immunohistochemical Staining

Selected blocks were cut into 4 μm-thick sections and placed on positively charged slides. The prepared sections were then immunohistochemically stained using the PD-L1 antibody (22C3 clone, 1/50; Dako A/S, Glostrup, Denmark). This procedure was performed using the Dako Omnis fully automated immunohistochemical staining device system (Dako A/S, Glostrup, Denmark) and the EnVision FLEX system (Acilent Technologies, Inc., Santa Clara, CA, USA). DAB (3,3’diamino-benzidine) was used as a chromogen. Positive and negative controls produced using the cell line method were used.

### Molecular Examination

*EGFR* mutation analysis was run on a fully automated system (*Cobas 4800; Roche Diagnostics, Basel, Switzerland*) using a real-time polymerase chain reaction. Exons 18, 19, 20, and 21 of the gene were evaluated. The fluorescence in situ hybridization (FISH) method was used for echinoderm microtubule-associated protein-like 4-anaplastic lymphoma kinase (*EML4-ALK*) translocation using the ZytoLight SPEC ALK and ROS1 Dual Color Break Apart Probe (*ZytoVision GmbH, Bremerhaven, Germany*). The signal pattern was evaluated in at least 50 tumor cells as standard procedure. If ≥15% split signals were detected, the test was considered to have yielded a positive result.

### Evaluation of Tumor-Infiltrating Lymphocytes (TIL)

**Figure 1:**
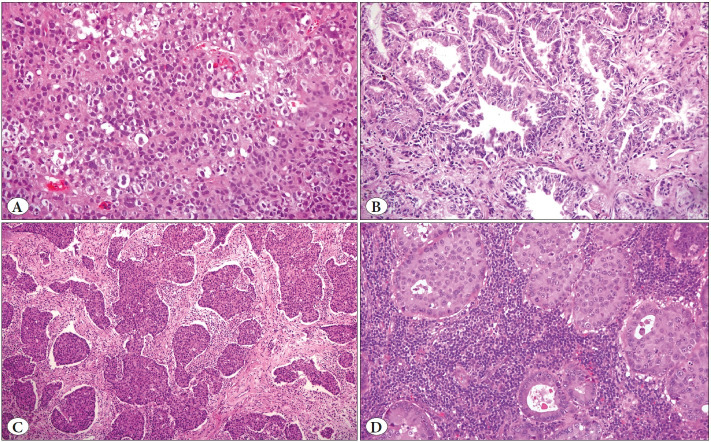
**A)** No lymphocytic response in tumor cells; TIL score 0 (Hematoxylin and eosin, x200). **B)** Scarce number of lymphocytes dispersed in the stroma; TIL score 1 (Hematoxylin and eosin, x200). **C)** Moderately dense presence of lymphocytes; TIL score 2 (Hematoxylin and eosin, x100). **D)** Diffuse presence of lymphocytes in the stroma and lymphocytes visible in tumor cells; TIL score 3 (Hematoxylin and eosin, x200).

Slides of the resection and biopsy specimens stained with hematoxylin and eosin (H&E) were evaluated for TIL. This assessment was performed using the scoring system published by the University of Pittsburgh (3,4). The assessment of TIL was divided into 4 categories: **Score 0:** No lymphocytic response in the tumor; **Score 1:** Scattered lymphocytic infiltration in the stroma, without intratumoral infiltration; **Score 2:** Moderately intense lymphocytic infiltration in the stroma, without intratumoral infiltration; **Score 3:** Diffuse lymphocytic infiltration in the stroma and appearance of lymphocytes in tumor cells (Figure 1). Crush artifacts and areas of necrosis or inflammation were not evaluated. The scores were grouped as TIL-negative (0-1) and TIL-positive (2-3).

### Evaluation of Immunohistochemical Biomarkers

**Figure 2:**
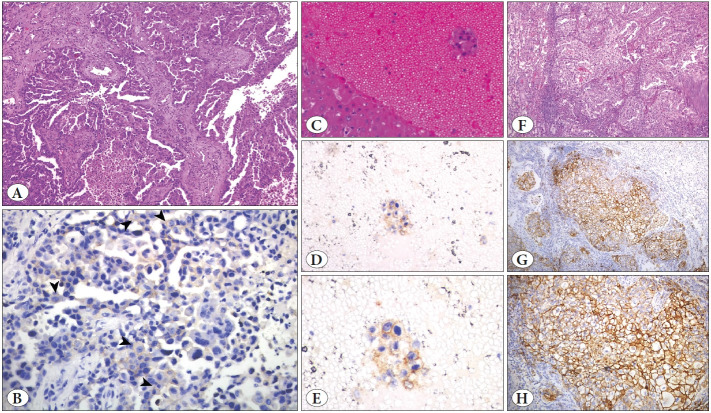
**A)** Metastatic pulmonary adenocarcinoma in the brain parenchyma (H&E, x100). **B)** Weak, incomplete membranous staining, arrowheads: Score 1 (PDL-1 x400). **C)** Metastatic pulmonary adenocarcinoma in the liver parenchyma, a cell block of FNAB (H&E, x200). **D,E)** Moderate complete and incomplete membranous staining; Score 2+ (PD-L1, x200 and x400). **F)** Resection material of a case of pulmonary adenocarcinoma (H&E, x100). **G,H)** Strong, complete membranous staining; Score 3+ (PD-L1, x100 and x200).

H&E-stained slides of the same section were evaluated by 2 observers using a CX40 microscope (Olympus Corp., Tokyo, Japan). Macrophages, which may mimic tumors by staining positively, particularly in cell block preparations, were morphologically identified and positive staining in these cells was ignored. PD-L1 expression in tumor cells was assessed under x20 and x40 magnification and scored as described in Table 1. Only membranous staining was evaluated, as suggested in previous studies and guidelines, and cytoplasmic staining were not considered as a positive result (5). Membranous staining was grouped according to intensity (Figure 2) and whether the expression was complete or incomplete. The tumor proportion ratio was calculated (6,7). Stained areas with diffuse necrosis or tissue edge artifacts were not considered as a positive stain.

**Table 1:** Parameters used to evaluate PD-L1 expression in tumor cells.

**Presence of PD-L1 expression (membranous)**	**PD-L1 expression intensity**	**PD-L1 expression** **pattern**	**PD-L1-expressing tumor cell** **/ total tumor cell (%)**
**Positive**	**1+**	**Complete**	**<1%**
**Negative**	**2+**	**Incomplete**	**1-9%**
	**3+**		**10-49%**
			**≥50%**

**PD-L1:** Programmed cell death ligand-1.

### Statistical Analysis

When evaluating the findings obtained in this study, IBM SPSS Statistics for Windows, Version 22.0 (IBM Corp., Armonk, NY, USA) was used to perform statistical analyses. The Shapiro-Wilk test was used in order to check whether the distribution of the variables was normal or not. In addition to descriptive statistical methods (mean, standard deviation, frequency), the one-way Anova test was used for comparisons of parameters between more than two groups, and Student’s t-test was used for comparisons between two groups. A chi-square test, Fisher’s exact chi-square test, continuity (Yates) correction, and the Fisher-Freeman-Halton test were used for the comparison of qualitative data. Kaplan-Meier analysis and the log-rank test were used for survival analysis. Significance was assessed at p<0.05.

## RESULTS

### Data Concerning Molecular Studies

There were 337 patients with NSCLC who had undergone molecular analyses and were diagnosed based on the evaluation of the cytology cell block, small biopsy, or resection material during the study period. Of these, 177 consultation cases, 24 cases with an inadequate number of cells, and 14 cases in which the slides and paraffin blocks were not accessible from the archive were excluded. A total of 122 cases with NSCLC were included in the study. The characteristics of the cases are summarized in Table 2.

**Table 2:** Study parameters.

		**n**	**%**
Site	Lung	63	51.6
	Extrapulmonary sites	59	48.4
Organ	Lung	63	51.6
	Brain	26	21.3
	Skin/subcutaneous tissue	3	2.5
	Liver	2	1.6
	Bone	3	2.5
	Lymph node	12	9.8
	Pericardium	3	2.5
	Pleura	8	6.6
	Adrenals	2	1.6
Sampling method	Biopsy	26	21.3
	Resection	63	51.6
	Cell block	33	27
Histopathological Diagnosis	Lung adenocarcinoma	106	86.9
	Large cell neuroendocrine carcinoma	2	1.6
	Undifferentiated large cell carcinoma	4	3.3
	NSCLC, without special type	8	6.6
	Pleomorphic carcinoma	1	0.8
	Squamous cell carcinoma	1	0.8
*EGFR *mutation status	Positive	24	19.7
	Negative	98	80.3
*EGFR* mutation site	Exon 19 Exon 21 Exon 18 Exon 20	9 9 3 3	37.5 37.5 12.5 12.5
*ALK* mutation status	Invalid	4	3.3
	Negative	49	40.2
	None	69	56.6
*ROS1* mutation status	Invalid	6	4.9
	Negative	18	14.8
	None	98	80.3
Cell count	<100	18	14.8
	>100	104	85.2

### Immunohistochemical Evaluation Results of PD-L1 in the Tumor

PD-L1 staining was not observed in 87. In 11 cases, less than 1% staining was recorded. As a result, 98 cases (80.3%) were considered PD-L1-negative, and 24 cases (19.7%) were regarded as PD-L1-positive. In 9 (7.4%) of these cases, the positivity rate was ≥50%. The staining intensity was 1+ in 6 (25%), 2+ in 11 (45.8%), and 3+ in 7 (29.2%) PD-L1-positive cases. Among the PD-L1-positive cases, complete staining was seen in 15 (62.5%) and incomplete staining was observed in 9 (37.5%) cases (Table 3).

**Table 3:** Immunohistochemical evaluation results of PD-L1 in the tumor.

		**n**	**%**
PD-L1	Negative	98	80.3
	Positive (≥1%)	24	19.7
	Negative	98	80.3
	Positive (<50%)	15	12.3
	Positive (≥50%)	9	7.4
PD-L1 staining intensity (n=24)	1	6	25
	2	11	45.8
	3	7	29.2
PD-L1 characteristics (n=24)	Complete	15	62.5
	Incomplete	9	37.5

**PD-L1:** Programmed cell death ligand-1.

### Presence of Tumor-Infiltrating Lymphocytes

Once cell blocks, lymph node metastases, and cases with very extensive necrotic areas were excluded, the intensity of TIL was evaluated in 80 cases. In all, 13 cases were assessed as having a TIL score of 0, and 40 cases as having a TIL score of 1. A total of 53 cases (66.3%) were accepted as TIL-negative. Eighteen cases and 9 cases were evaluated as TIL score 2+ and TIL score 3, respectively. A total of 27 cases (33.8%) were accepted as TIL-positive.

### The Results Obtained When Positive PD-L1 Expression was Defined at Tumor Proportion Score Values Equal to or Greater Than 1%

There was a statistically significant correlation between PD-L1 positivity and *EGFR* mutation status (p=0.042). Only 1 (4.16%) of the 24 cases with *EGFR*-positivity had PD-L1 positivity. In all, 23 cases (95.83%) with *EGFR*-positivity were PD-L1-negative. There was no statistically significant difference between PD-L1-positive and negative cases in terms of the other parameters. (p>0.05) (Table 4).

**Table 4:** The results obtained when positive programmed cell death ligand-1 expression was defined at a tumor proportion score value equal to or exceeding 1%.

		**PD-L1-positive**	**PD-L1- negative**	**p**
Age (mean ± SD)		60.33±8.33	62.42±10.44	^1^0.365
Gender, n (%)	Male	19 (20.7)	73 (79.3)	^2^0.832
	Female	5 (16.7)	25 (83.3)	
Site, n (%)	Lung	12 (19)	51 (81)	^2^1.000
	Others	12 (20.3)	47 (79.7)	
Organ	Lung	12 (19)	51 (81)	^4^0.698
	Brain	5 (19.2)	21 (80.8)	
	Lymph node	1 (8.3)	11 (91.7)	
	Pericardium-pleura	3 (27.3)	8 (72.7)	
	Others	3 (30)	7 (70)	
Sampling method	Biopsy	5 (19.2)	21 (80.8)	^5^0.958
	Resection	13 (20.6)	50 (79.4)	
	Cytological cell block	6 (18.2)	27 (81.8)	
Histopathological diagnosis	Pulmonary adenocarcinoma	20 (19.8)	81 (80.2)	^3^1.000
	Others	4 (19)	17 (81)	
EGFR mutation status, n (%)	Positive	1 (4.2)	23 (95.8)	^3^0.042*
	Negative	23 (23.5)	75 (76.5)	
Presence of TIL (n=80), n (%)	Positive	9 (33.3)	18 (66.7)	^2^0.170
	Negative	9 (17)	44 (83)	
Number of cells, n (%)	<100	3 (16.7)	15 (83.3)	^3^1.000
	>100	21 (20.2)	83 (79.8)	

^1^Student’s t-test, ^2^Continuity (Yates) correction, ^3^Fisher’s exact test, ^4^Fisher-Freeman-Halton test, ^5^Chi-square test; * p<0.05. **EGFR:** Epidermal growth factor receptor, **PD-L1:** programmed cell death ligand-1, **TIL:** tumor-infiltrating lymphocytes.

### Results of PD-L1 Expression in Tumor cells at Tumor Proportion Score of More or Less Than 50%

There was no statistically significant difference between the parameters (Table 5).

**Table 5:** The results obtained when positive programmed cell death ligand-1 expression was defined at a tumor proportion score value equal to or exceeding 50%.

		**PD-L1**			**p**
		**<50%**	**≥50%**	**Negative**	
Age (mean ± SD)		61.73±9.44	58.0±5.81	62.42±10.44	^1^0.453
Gender, n (%)	Male	12 (13)	7 (7.6)	73 (79.3)	^2^0.925
	Female	3 (10)	2 (6.7)	25 (83.3)	
Site, n (%)	Lung	9 (14.3)	3 (4.8)	51 (81)	^2^0.466
	Other	6 (10.2)	6 (10.2)	47 (79.7)	
Organ	Lung	9 (14.3)	3 (4.8)	51 (81)	^3^0.730
	Brain	3 (11.5)	2 (7.7)	21 (80.8)	
	Lymph node	0 (0)	1 (8.3)	11 (91.7)	
	Pericardium-Pleura	2 (18.2)	1 (9.1)	8 (72.7)	
	Other	1 (10)	2 (20)	7 (70)	
Sampling method	Biopsy	4 (15.4)	1 (3.8)	21 (80.8)	^2^0.904
	Resection	7 (11.1)	6 (9.5)	50 (79.4)	
	Cytological Cell block	4 (12.1)	2 (6.1)	27 (81.8)	
Histopathological diagnosis	Pulmonary adenocarcinoma	12 (11.9)	8 (7.9)	81 (80.2)	^2^0.905
	Other	3 (14.3)	1 (4.8)	17 (81)	
*EGFR* mutation status, n (%)	Positive	1 (4.2)	0 (0)	23 (95.8)	^2^0.125
	Negative	14 (14.3)	9 (9.2)	75 (76.5)	
Presence of TIL (n=80), n (%)	Positive	5 (18.5)	4 (14.8)	18 (66.7)	^2^0.234
	Negative	6 (11.3)	3 (5.7)	44 (83)	
Number of cells, n (%)	<100	3 (16.7)	0 (0)	15 (83.3)	^2^0.437
	>100	12 (11.5)	9 (8.7)	83 (79.8)	

^1^One-way analysis of variance test; ^2^Fisher-Freeman-Halton test; ^3^Chi-square test; * p<0.05. **EGFR:** Epidermal growth factor receptor, **PD-L1:** programmed cell death ligand-1, **TIL:** tumor-infiltrating lymphocytes.

### Results When PD-L1 Expression in Tumor Cells was Grouped According to Tumor Proportion Score <1%, 1-9%, 10-49%, and ≥50%

The tumor proportion score significantly differed between TIL positive and TIL negative groups (p=0.016). No statistically significant correlation was detected among the other parameters (Table 6).

**Table 6:** Programmed cell death ligand-1 expressions in tumor cells grouped according to tumor proportion score.

		**PD-L1**					**p**
		**0**	**<1%**	**1-9%**	**10-49%**	**%50+**	
Age (mean ± SD)		62.32±10.79	63.18±7.45	61.22±10.02	62.5±9.35	58.0±5.81	^1^0.791
Gender, n (%)	Male	66 (75.9)	7 (63.6)	8 (88.9)	4 (66.7)	7 (77.8)	^2^0.738
	Female	21 (24.1)	4 (36.4)	1 (11.1)	2 (33.3)	2 (22.2)	
Sampling site, n (%)	Lung	45 (51.7)	6 (54.5)	5 (55.6)	4 (66.7)	3 (33.3)	^2^0.765
	Others	42 (48.3)	5 (45.5)	4 (44.4)	2 (33.3)	6 (66.7)	
Sampled organ	Lung	45 (51.7)	6 (54.5)	5 (55.6)	4 (66.7)	3 (33.3)	^2^0.640
	Brain	18 (20.7)	3 (27.3)	3 (33.3)	0 (0)	2 (22.2)	
	Lymph node	6 (6.9)	1 (9.1)	1 (11.1)	0 (0)	2 (22.2)	
	Pericardium-pleura	10 (11.5)	1 (9.1)	0 (0)	0 (0)	1 (11.1)	
	Other	8 (9.2)	0 (0)	0 (0)	2 (33.3)	1 (11.1)	
The sampling method	Biopsy	20 (23)	1 (9.1)	3 (33.3)	1 (16.7)	1 (11.1)	^2^0.142
	Resection	40 (46)	10 (90.9)	5 (55.6)	2 (33.3)	6 (66.7)	
	Cytological cell block	27 (31)	0 (0)	1 (11.1)	3 (50)	2 (22.2)	
Histopathological diagnosis of the sample	Pulmonary adenocarcinoma	71 (81.6)	10 (90.9)	6 (66.7)	6 (100)	8 (88.9)	^2^0.446
	Other	16 (18.4)	1 (9.1)	3 (33.3)	0 (0)	1 (11.1)	
*EGFR* mutation status, n (%)	Positive	22 (25.3)	1 (9.1)	1 (11.1)	0 (0)	0 (0)	^2^0.158
	Negative	65 (74.7)	10 (90.9)	8 (88.9)	6 (100)	9 (100)	
Presence of TIL (n=80), n (%)	Positive	11 (21.2)	7 (70)	4 (50)	1 (33.3)	4 (57.1)	^2^0.016*
	Negative	41 (78.8)	3 (30)	4 (50)	2 (66.7)	3 (42.9)	
Number of cells, n (%)	<100	15 (17.2)	0 (0)	2 (22.2)	1 (16.7)	0 (0)	^2^0.366
	>100	72 (82.8)	11 (100)	7 (77.8)	5 (83.3)	9 (100)	

^1^Student’s t-test; ^2^Chi-square test. * p<0.05. **EGFR:** Epidermal growth factor receptor, **PD-L1:** programmed cell death ligand-1, **TIL:** tumor-infiltrating lymphocytes.

### Overall Survival

Ninety of the 122 patients (73.8%) died. The longest and the shortest follow-up period was 56 months and 9 months, respectively. The cases were followed up by the oncology department of the hospital, and they were receiving the standard systemic treatment according to the National Comprehensive Cancer Network guidelines. The most recent death occurred in the 39th month of the follow-up period, with a cumulative survival rate of 13.8% and a standard error of 4.9%.**

**Figure 3:**
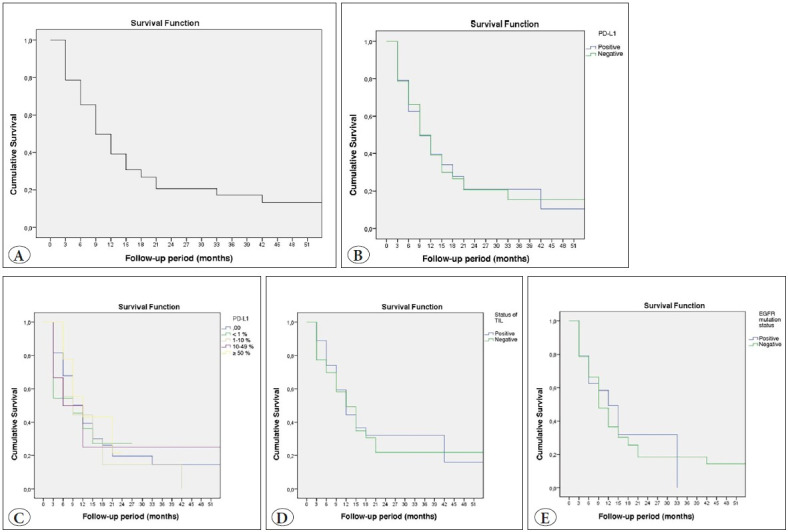
**A)** Overall survival. **B)** Overall survival rates for PD-L1-positive cases. **C)** Survival rates based on programmed cell death ligand-1 level. **D)** Overall survival rate based on tumor-infiltrating lymphocytes. **E)** Overall survival rate of cases according to epidermal growth factor receptor mutation.

**The survival time ranged from 8 days to 55 months (mean±SD: 16.13±1.88 months) (median survival time, 8.6 months). The cumulative survival rate at 3 and 6 months, and 1, 2, and 4 years was 66%, 50%, 31%, 21%, and 13%, respectively**. **None of the deaths occurred within the first month after diagnosis due to surgical intervention (Figure 3).

### Overall Survival Analysis When Positive PD-L1 Expression was Defined at Tumor Proportion Score of ≥1%

Eighteen (75%) of the 24 PD-L1-positive patients died during the study period. The last death occurred at the 39th month of follow-up. The survival rate was 10.2% with a standard error of 8.7%. The mean length of survival was 15.6±3.83 months. The cumulative survival rate at 3 and 6 months, and at 1, 2, and 4 years was 63%, 50%, 34%, 21%, and 10%, respectively. Seventy-two (73.5%) of the 98 PD-L1-negative patients died. The last death was seen at the 30th month of the follow-up period, yielding a survival rate of 15.7% with a standard error of 5.8%. The mean survival time was 16.37±2.22 months. The cumulative survival rate at 3 and 6 months, and at 1, 2, and 4 years was 66%, 50%, 30%, 21%, and 15%, respectively. No statistically significant difference was found between monthly survival rates according to PD-L1-positivity (p=0.873, p>0.05) (Figure 3). There was also no statistically significant difference (p=0.920, p>0.05) in the survival rate based on PD-L1 level where the PD-L1 expression in tumor cells was grouped according to a tumor proportion score of <1%, 1-9%, 10-49%, and ≥50%, when evaluated using the log-rank test (Figure 3).

### Correlation Between Tumor-Infiltrating Lymphocytes and Overall Survival

Nineteen (70.4%) of the 27 TIL-positive cases died. The last death occurred at the 17th month of the follow-up period. The survival rate was 31.7%, with a standard error of 9.4%. The mean survival time was 19.99±3.96 months. The cumulative survival rate at 3 and 6 months, and 1, 2, and 4 years was 74%, 59%, 37%, 32%, and 16%, respectively. Thirty-four (64.2%) of the 53 TIL-negative patients died. The most recent death was seen at the 19th month of the follow-up period, and the survival rate was 22.5% with a standard error of 7.6%. The mean survival time was 19.05±3.32 months. The cumulative survival rate at 3 and 6 months, and 1, 2, and 4 years was 70%, 58%, 35%, 22%, and 22%, respectively. There was no statistically significant difference (p=0.920, p>0.05) in the survival rate based on the presence of TIL, when evaluated using the log-rank test (p=0.851, p>0.05) (Figure 3).

### Correlation Between Epidermal Growth Factor Receptor Mutation Status and Overall Survival

Sixteen (66.7%) of the 24 patients with *EGFR*-positivity died. The most recent death was seen at the 30th month of the follow-up period, with a survival rate of 0%. The mean survival time was 14.84±2.60 months. The 3- and 6-month, and 1- and 2-year cumulative survival rate was 63%, 58%, 32%, and 24%, respectively. Seventy-four (75.5%) of the 98 *EGFR*-negative patients died. The last death occurred at the 39th month of the follow-up period. The survival rate was 14.5%, with a standard error of 4.9%. The mean length of survival was 15.87±2.03 months. The cumulative survival rate at 3 and 6 months, and at 1, 2, and 4 years was 66%, 48%, 30%, 18%, and 14%, respectively. There was no statistically significant difference between the survival rate of *EGFR*-positive and *EGFR*-negative cases (p=0.537), when evaluated using the log-rank test (Figure 3).

## DISCUSSION

Lung cancer is the leading cause of cancer-related death in the world. Each year it causes the death of approximately 1.6 million people. This estimate is greater than the annual sum of breast, colon, and prostate cancer-related deaths (8). NSCLC constitutes most of the lung cancer diagnoses, and most patients are metastatic at the time of diagnosis (9). Despite improved overall survival with the classic platinum-based chemotherapies, survival in patients with advanced NSCLC remains at approximately 8 to 12 months (10,11). As a result of the use of molecular assays in NSCLC, and especially studies of the adenocarcinoma subtypes, driver genetic mutations that can be used for targeted therapies have gradually been recognized (12). Targeted therapies for *EGFR, ALK*, and *ROS1* have been approved, and studies continue for *MET, HER2, BRAF, RET, NTRK1, MEK1*, and *PIK3CA* gene mutations. Despite the success of targeted therapies, the development of treatment resistance and the progression of the disease constitute major problems (13). In patients who have no driver mutation and those who have been treated with chemotherapy, the treatment approaches to be applied in case of disease progression are much more limited. All these reasons have made it necessary to find new options for the treatment of lung cancer. One of these is immunotherapy.**

**Immunotherapy, in its simplest form, can be defined as any treatment that interacts with the immune system to treat cancer. Studies have also gained momentum with the discovery of immune checkpoint inhibitors, one of the main mechanisms by which the tumor escapes the immune response. So far, 2 important immune checkpoints with established effectiveness in cancer treatment have been investigated: the CTLA-4 pathway and the PD-1/PD-L1 pathway (2). The use of the CTLA-4 inhibitor ipilimumab was first approved by the US Food and Drug Administration (FDA) for melanoma in 2011 (14,15). This drug was found to provide a survival benefit in squamous cell carcinoma in studies performed with NSCLC patients, which was subsequently followed by phase III trials (16). In the PD1/PD-L1 pathway, there are PD-1 inhibitors such as nivolumab and pembrolizumab, and PD-L1 inhibitors such as atezolizumab. In the KEYNOTE-010 trial published in 2016, advanced NSCLC cases with at least 1% PD-L1-positivity in the tumor were included in the study and treated with pembrolizumab. The results showed an increase in survival time when compared with chemotherapy. Survival was significantly longer (8.2 months vs. 17.3 months) when the expression of PD-L1 in the tumor was greater than 50%. The same study also indicated that the treatment-related toxicity was lower than with standard chemotherapy (17). In the KEYNOTE-024 trial published in 2017, patients with advanced stage NSCLC with tumor PD-L1 expression of 50% or more were randomized to receive pembrolizumab or platinum-based chemotherapy. Pembrolizumab had a higher rate of progression-free survival and treatment response with a lower incidence of side effects. This study demonstrated that anti-PD-L1 therapy was superior in comparison with platinum-based chemotherapy in selected cases and led to FDA approval for first-line treatment with the drug in patients with advanced NSCLC (18). Considering all these developments, the selection of patients who can benefit from these drugs via the accurate detection of PD-L1-positive tumors becomes important. The literature suggests that immunofluorescence, enzyme-linked immunosorbent assay (ELISA), and real-time polymerase chain reaction techniques are very rarely used in the evaluation of PD-L1. The most frequently used and most widely accepted method is immunohistochemical analysis (19–21).

In our results, there was no significant difference in the incidence of PD-L1-positive or -negative tumors in terms of age or gender. Some studies in the literature have suggested that PD-L1 expression is seen more frequently at a young age (22,23); however, Brody et al. (24) reported no significant relationship between age and gender and PD-L1 in a large meta-analysis.**

**Most studies of PD-L1 in lung cancer have been evaluated in the primary organ. In our study, the presence of a sufficient quantity of specimens from biopsies of both the primary tumor and the metastases enabled us to make a comparison in terms of positivity. Approximately half of the PD-L1-positive cases were from the lung and the other half were from extrapulmonary metastases. The positivity rate was almost equal (p=1.00). When the cases were evaluated according to the organ, no significant difference was found in terms of positivity.

There are a small number of studies in the literature that have evaluated the relationship between the metastasis site and PD-L1-positivity in advanced tumors. In a study that was like ours in terms of methodology but with a smaller number of cases, the results were consistent with our results (25). In another study, PD-L1 was evaluated using an ELISA in tumor cells circulating in the blood, and antibody positivity was significantly higher in abdominal organ metastases. In a study comparing brain and other organ metastases in EGFR-positive patients with metastatic tumors who received a tyrosine kinase inhibitor (TKI), no significant difference in PD-L1-positivity was found (21,26).

In our study, PD-L1 IHC was examined in resection, biopsy, and cytology cell block specimens, and PD-L1-positivity rates were compared. The positive and negative rates were similar in all 3 groups and there was no statistically significant difference between them (p=0.958). There is only a limited number of studies evaluating such cytology samples in the literature.**

**It has recently been reported, particularly in histology-cytology publications, that stains of cytological specimens were compatible with tissue stains (27–29). Interestingly, Heymann et al. (27) have found the rate of PD-L1 expression in cytology specimens to be greater than that in biopsy and resection material, and suggested that this result might be related to cytology specimens obtained from patients with a more advanced stage of disease. These studies and our research demonstrate the utility of PD-L1 IHC in cytology samples, which can be obtained in minimally invasive and easily applicable procedures.

Targeted therapies and tumor subtyping for anti-PD-1/PD-L1 treatments are important, but it is not always possible to distinguish adenocarcinoma from non-adenocarcinoma in advanced stage tumors, especially when only a small amount of tissue is available. In our study, 21 cases were diagnosed with non-adenocarcinoma NSCLC. Of these diagnoses, 4 were PD-L1-positive and 1 (a case diagnosed as pleomorphic carcinoma) had ≥50% positivity, it is noteworthy that the positivity rates in this group (19%) and that of adenocarcinoma (19.8%) were almost the same, although the number of cases was small. In the literature, there are publications that have detected very similar rates of positivity when comparing cases with adenocarcinoma and squamous cell carcinoma (30); however, PD-L1-positivity has also been significantly associated with adenocarcinoma (31).

In our study, the threshold value for PD-L1 expression was accepted as 1% or more, and the expression rate of PD-L1 in tumor cells was found to be 19.7%. In the literature, studies using the same clone have reported a very wide range from 4% to 66% at the same threshold (7,17,23–25,32). If the positivity threshold was accepted as ≥50%, the PD-L1 expression rate was 7.4%. When we look at these threshold values, very similar (23), higher (7,17,25), and lower rates (32) have been reported in the literature. These differences in PD-L1 expression may be due to differences in assessments (localization and percentage of staining, etc.), variety in patient populations, preanalytical phases, and material types. FDA-approved studies involving some clones other than 22C3 and performed mostly in advanced-stage patients have set this threshold at 5% (33,34), while others, similar to our study, have used a threshold of 1% for positivity (35–39). The reported positivity rate has ranged from 23% to 59%.

Although the expression of PD-L1 in tumor cells is theoretically expected to be higher in advanced cancer (40–42), no correlation between stage of disease and PD-L1 has been detected in most studies (23,40–43). Since our study was based on only advanced-stage cases, it is not possible to express an opinion about whether PD-L1 expression differs from stage to stage. Garon et al. (7) and Herbst et al. (17), who evaluated the 22C3 antibody in a large series of advanced stage NSCLC patients using a threshold value of 1%, obtained positivity rates of 61% and 66%, respectively. These rates are quite high compared with our results. One reason may be the ethnic origin of the patients. In 2 dissertation studies performed in Turkey (non-published data), the PD-L1-positivity rate was 22.8% and 9.1% (44,45). In these studies, an SP142 clone was used, and they selected a threshold value of 5%. Resection material was used, and patients from each disease stage were included; however, there was no significant difference between their results and ours.

The biomarkers of *EGFR* mutations and PD-L1 positivity have their own specific treatment approaches. There is no consensus on which treatment should be administered if EGFR- and PD-L1-positivity coexist. Combination therapy with these 2 drugs targeted to these biomarkers also requires a more careful assessment, due to the increased risk of toxicity compared with the early results of some studies (46). The first publications related to this issue argued that the *EGFR* mutation enhances PD-L1-positivity (43,47,48). The use of EGFR-TKI in *EGFR*-mutant tumors has been shown to reduce PD-L1 receptors on the cell membrane and not on “wild types,” suggesting a role of EGFR mutation in PD-L1 expression. In a mouse study, anti-PD1 treatment of *EGFR*-mutant lung tumors has shown that these 2 pathways interact with each other by stopping tumor growth and increasing survival (47). The advocated view in that article is that EGFR-TKIs will increase survival by suppressing both EGFR signaling and PD-L1 expression. When combined with anti-PD-1 therapy, the response will be increased, and resistance will be reduced. D’Incecco et al. (31) also found that PD-L1-positivity was significantly associated with *EGFR* mutation in advanced NSCLC. Aside from these findings, there was no significant association between EGFR and PD-L1 in later studies, especially in early-stage tumors (23,30,48–51). In a group study involving stage IV patients, PD-L1-positivity was found to be associated with *EGFR* “wild type” (25,52–55), contrary to earlier results (Table 7).

**Table 7:** Studies demonstrating correlations between epidermal growth factor receptor mutation status and programmed cell death ligand 1 expression.

**Author**	**Tumor histology**	**Stage**	**Cases, n**	**Relationship between EGFR and PD-L1**	**PD-L1 Antibody**
Azuma (43)	NSCLC	I-III	164	Higher rate of PD-L1- positivity in EGFR mutants	Mouse mAb
D’Incecco (31)	NSCLC	IV	125	Higher rate of PD-L1- positivity in EGFR mutants	ab58810
Koh (49)	Adenocarcinoma	I-III	497	Unrelated	E1L3N
Cooper (23)	NSCLC	I-III	681	Unrelated	22C3
Schmidt (30)	NSCLC	I-III	321	Unrelated	E1L3N
Tsao (51)	NSCLC	I-III	982	Unrelated	E1L3N
Tang (48)	NSCLC	IIIB-IV	170	Unrelated	E1L3N
Mori (50)	NSCLC	I-III	296	Unrelated	EPR1611
Ji (54)	Adenocarcinoma	I-III	100	Higher rate of PD-L1- positivity in EGFR-wild type	Ab174838
Takada (55)	Adenocarcinoma	I-III	417	Higher rate of PD-L1- positivity in EGFR-wild type	SP142
Inamura (53)	Adenocarcinoma	I-IV	268	Higher rate of PD-L1- positivity in EGFR-wild type	E1L3N
Huynh (52)	Adenocarcinoma	I-IV	261	Higher rate of PD-L1- positivity in EGFR-wild type	E1L3N
Rangachari (25)	Adenocarcinoma	I-IV	71	Higher rate of PD-L1- positivity in EGFR-wild type	22C3
Present study	NSCLC	IV	122	Higher rate of PD-L1- positivity in EGFR-wild type	22C3

**EGFR:** Epidermal growth factor receptor, **NSCLC:** non-small cell lung carcinoma, **PD-L1:** programmed cell death ligand 1.

In our study, there was a statistically significant relationship between PD-L1-positivity and *EGFR* mutation status (p=0.042). Only 1 of the 24 *EGFR*-positive cases (4.16%) had PD-L1 positivity. According to our results, these 2 biomarkers have almost mutually exclusive positives. This relationship is consistent with the results of recent studies in the literature performed with advanced-stage cases.

Differences in sample size in the studies; potential bias due to retrospective collection of data; heterogeneity of some basic patient characteristics such as pathological stage and racial differences; different PD-L1- positivity thresholds; and variations in antibody and immuno-technical issues may be reasons for the differences in the results in *EGFR* and PD-L1 studies, as well as variability in the percentages of positivity.**

**In our study, a statistically significant correlation was detected between tumor positivity and the presence of TIL, and with PD-L1-staining in TIL (+) cases (p=0.016)**

**The presence of TIL in tumors with negative PD-L1 staining was significantly lower than in other groups. Although this emerging significance complies with the theory of acquired immune resistance in the tumor microenvironment (2), further studies with larger samples are needed.

In the evaluation of PD-L1, at least 100 tumor cells are needed for clones 28-8 and 22C3, and at least 50 tumor cells for SP142 clones (5). In our study, at least 100 tumor cell-containing tissues were initially included in the study; however, 18 slides prepared for immunohistochemical analysis of PD-L1 contained less than 100 tumor cells, and this group was evaluated separately. There was no significant difference between the 2 groups for PD-L1-positivity.

According to this result, we recommend performing a PD-L1 immunohistochemical study even if there are not enough cells (following diagnostic immunohistochemical examination and molecular analysis). In the event of a negative outcome, excision of a new tissue sample may be an option.

A total of 90 of the 122 patients (73.8%) in the study died. The most recent death was in the 39th month, with a cumulative survival rate of 13.8% and a standard error of 4.9%. The mean survival time ranged from 8 days to 55 months with a mean survival time of 16.13±1.88 months. The cumulative survival rate at 3 and 6 months, and 1, 2, and 4 years of the follow-up period was 66%, 50%, 31%, 21%, and 13%, respectively. These values are consistent with the results found in large series (56).**

**In phase III trials performed with metastatic lung cancer patients with EGFR mutations, patients treated with EGFR TKI did not demonstrate a significant change in overall survival despite a high response rate and longer progression-free survival (57). Driver mutations and targeted therapies are increasingly important and promising methods in recent years, but there was no statistically significant difference between the overall survival of *EGFR*-positive and negative cases (p=0.537) in our results. Together with this result, *EGFR*-positivity was not a confounding variable in PD-L1-positive and negative group survival analysis, and it increased the reliability of the results. There was no statistically significant difference between PD-L1-positive and negative cases in terms of survival analysis (p>0.05). The literature reports indicate that PD-L1-positivity has been associated with both shorter (21,40) and longer survival time (20,23), while in some studies, consistent with our results, PD-L1-positivity was not associated with survival time (30,41). In most studies, heterogeneous groups, including early-stage cases, were evaluated. These conflicting results demonstrate the need for controlled, randomized trials in homogeneous groups with more cases in order to determine the prognostic value of PD-L1.

One of the limitations of this study is that driver mutations other than *EGFR* (*ALK* and *ROS1*) were not included in the statistical analysis because the number of cases was small and there were no positive cases. Another limitation may be the relatively small number of cases with the presence of TIL. There is a need for comparative studies where the technique, clones, threshold values, and phases are homogenized.

## Conflict of Interest

The authors declare no conflict of interest.
